# Severe suicide attempts in young adults: suicide intent is correlated with medical lethality

**DOI:** 10.1590/S1516-31802005000100011

**Published:** 2005-01-02

**Authors:** Claudemir Benedito Rapeli, Neury José Botega

Suicidal behavior is currently thought of as a continuum. Although there is a wide variety of such behavior, most of the information for the construction of epidemiological standards is still derived from statistics of death by suicide. However, the burden associated with suicide attempts represented 1.4% of the overall burden of diseases in 2002. It is estimated that this figure will reach 2.4% in 2020.^1^

Our study evaluated 121 patients who had been hospitalized because of medically severe suicide attempts, at Hospital das Clínicas of the Universidade Estadual de Campinas. Beck’s Suicide Intent Scale (IS) was used for measuring the patient’s intention of dying. The degree of lethality was evaluated on a six-point scale, based on the patient’s clinical status and also on the procedures performed during hospitalization, clinical parameters, threat to life and the attending physician’s impressions. The Spearman correlation test was used, for all patients and according to age groups, to evaluate the correlation between suicidal intent and lethality.

There was a correlation between suicidal intent and lethality (correlation coefficient of 0.42; p = 0.04) within the 30 to 39-year-old group (38 individuals). Most of these patients were male (60%) and were married or living with someone (60%). 44% had a history of psychiatric treatment. Poisoning was the method most commonly used (88%) and 52% of the patients had the diagnostic hypothesis of affective disorder.

Previous studies have shown a correlation between lethality and intent, based on the assumption that the individuals are aware of the lethal potential of their suicide attempt.^2^ In our study, we strictly evaluated severe suicide attempts, carefully measuring suicidal intent and lethality.

We noted that, for at least one third of the patients, there was a correlation, albeit moderate, between the desire to die and the lethality of the method chosen for suicide. Therefore, the resulting injuries were severe and life-threatening. Some of the patients would probably have died, had they not been cared for in a hospital. In the future, would these patients be at higher risk of dying through suicide? If we take the scientific literature into account, the answer is yes.

Two important suicide predictors gather several risk factors. Firstly, previous suicide attempts: in Finland, a cohort study of 100 attempts was followed over 37 years, in the longest study of this type ever made, revealing a suicide rate of 31.2%.^3^ Secondly, suicide attempts resulting in severe injuries, posing threat to life: in a study in New Zealand with 302 individuals in this category, 7% died through suicide after 5 years.^4^

In Brazil, recent statistical data (comparison between the periods of 1980-82 and 1998-2000) show that the suicide coefficient has increased by 32.8% among men. In the municipality of São Paulo, for example, the young adult age group (25-44 years of age) accounts for 45% of all deaths due to suicide.^5^

In summary, our findings suggest that the subgroup of patients consisting of young adults with medically severe suicide attempts are at greater risk of a future suicide attempt. Thus, when identified by healthcare professionals, these patients need special attention. It is important to diagnose the patient’s living conditions as well as to establish a bond that enables treatment continuity.
